# Predicting EGFR and PD-L1 Status in NSCLC Patients Using Multitask AI System Based on CT Images

**DOI:** 10.3389/fimmu.2022.813072

**Published:** 2022-02-18

**Authors:** Chengdi Wang, Jiechao Ma, Jun Shao, Shu Zhang, Zhongnan Liu, Yizhou Yu, Weimin Li

**Affiliations:** ^1^ Department of Respiratory and Critical Care Medicine, Med-X Center for Manufacturing, National Clinical Research Center for Geriatrics, Frontiers Science Center for Disease-related Molecular Network, West China Hospital, West China School of Medicine, Sichuan University, Chengdu, China; ^2^ AI Lab, Deepwise Healthcare, Beijing, China; ^3^ Faculty of Engineering, The University of Hong Kong, Hong Kong, Hong Kong SAR, China

**Keywords:** EGFR, PD-L1, NSCLC, deep learning, computed tomography

## Abstract

**Background:**

Epidermal growth factor receptor (EGFR) genotyping and programmed death ligand-1 (PD-L1) expressions are of paramount importance for treatment guidelines such as the use of tyrosine kinase inhibitors (TKIs) and immune checkpoint inhibitors (ICIs) in lung cancer. Conventional identification of EGFR or PD-L1 status requires surgical or biopsied tumor specimens, which are obtained through invasive procedures associated with risk of morbidities and may be unavailable to access tissue samples. Here, we developed an artificial intelligence (AI) system that can predict EGFR and PD-L1 status in using non-invasive computed tomography (CT) images.

**Methods:**

A multitask AI system including deep learning (DL) module, radiomics (RA) module, and joint (JO) module combining the DL, RA, and clinical features was developed, trained, and optimized with CT images to predict the EGFR and PD-L1 status. We used feature selectors and feature fusion methods to find the best model among combinations of module types. The models were evaluated using the areas under the receiver operating characteristic curves (AUCs).

**Results:**

Our multitask AI system yielded promising performance for gene expression status, subtype classification, and joint prediction. The AUCs of DL module achieved 0.842 (95% CI, 0.825–0.855) in the EGFR mutated status and 0.805 (95% CI, 0.779–0.829) in the mutated-EGFR subtypes discrimination (19Del, L858R, other mutations). DL module also demonstrated the AUCs of 0.799 (95% CI, 0.762–0.854) in the PD-L1 expression status and 0.837 (95% CI, 0.775–0.911) in the positive-PD-L1 subtypes (PD-L1 tumor proportion score, 1%–49% and ≥50%). Furthermore, the JO module of our AI system performed well in the EGFR and PD-L1 joint cohort, with an AUC of 0.928 (95% CI, 0.909–0.946) for distinguishing EGFR mutated status and 0.905 (95% CI, 0.886–0.930) for discriminating PD-L1 expression status.

**Conclusion:**

Our AI system has demonstrated the encouraging results for identifying gene status and further assessing the genotypes. Both clinical indicators and radiomics features showed a complementary role in prediction and provided accurate estimates to predict EGFR and PD-L1 status. Furthermore, this non-invasive, high-throughput, and interpretable AI system can be used as an assistive tool in conjunction with or in lieu of ancillary tests and extensive diagnostic workups to facilitate early intervention.

## Introduction

Lung cancer is the second most commonly diagnosed cancer and the leading cause of mortality tumor throughout the world (1, 2). In China, there are around 733,000 new cases of lung cancer annually, and with over 610,000 deaths due to lung cancer (3), accounting for 37% new cases and 39.2% death cases of the world, respectively (4). Approximately 85% of lung cancer patients were histologically identified as non-small cell lung cancer (NSCLC), of which comprises the most common subtype such as lung adenocarcinoma (LUAD) and lung squamous cell carcinoma (LUSC) (5). Targeted therapies, as represented by epidermal growth factor receptor (EGFR) tyrosine kinase inhibitors (TKIs), and immune checkpoint inhibitor (ICI) treatments targeted the programmed death-1 (PD-1) receptor on T cells, or the programmed death ligand-1 (PD-L1) expressed by tumor cells; these two treatment paradigms have significantly revolutionized cancer treatment and improved survival outcome for lung cancer. Identifying predictive biomarkers is therefore crucial for choosing individuals who are potentially suitable to therapy.

In the era of precision medicine, lung cancer treatment depended on the genetics. Patients with EGFR mutated lung adenocarcinoma could achieve a longer progression-free survival (PFS) from EGFR-TKIs than conventional chemotherapy (6–8). However, the medication and efficacy varied among NSCLC patients with EGFR 19Del, L858R, or other types of mutations (9, 10). Meanwhile, ICIs targeting PD-1 or PD-L1 offer promising paradigm to treatment in NSCLC with high PD-L1 expression. The first-line pembrolizumab monotherapy can enhance overall survival (OS) and PFS in lung cancer patients with PD-L1 tumor proportion score (TPS) ≥50% (11, 12). However, gene detection is determined by surgical or biopsied tissue-based assays at present, which has many limitations: difficulties in accessing suitable tumor tissues due to their extensive genetic heterogeneity; associated morbidities or tumor metastasis during the invasive biopsies; and different antibodies, multiple scoring criteria, and poor DNA quality resulting in high heterogeneity of results (13). What is more, gene mutations could change over the course and progression during whole therapy, making it impractical and challenging to obtain tumor biopsy during multiple times. However, molecular profiling of relative high costs is not routinely performed for every patient, especially in low-resource settings. Therefore, a non-invasive method for identifying the mutation status is urgently needed.

Radiological images reflect abundant information on the entire tumor in non-invasive way (14). Recent advances in machine learning have promoted the disease diagnosis based on computed tomography (CT) images. Conventional radiomics methods, which are tedious and time consuming, include image segmentation, feature extraction and selection, model building, and data analysis. The radiological characteristics are affected by manual segmentation and CT scan parameters, and repeated professional analysis by doctors is necessary (14). Advances in deep learning could overcome these problems and have demonstrated accurate, reliable, and reproducible performance on triage tasks for detecting the abnormalities and diagnosing the disease (15, 16). These proposed deep learning models and techniques have achieved a predictive performance in estimating malignancy risk in pulmonary nodules and diagnosing pneumonia quickly during the COVID-19 pandemic. Recent new and exciting developments in artificial intelligence (AI) have provided new potential opportunities to predict the EGFR mutation or PD-L1 expression status on the basis of CT images (17, 18). However, the small datasets and binary task limit its applicability in the routine clinical work. There still exists a considerable challenge to objectively evaluate the ability of the model to predict the gene mutation status and gene subtypes.

In the present study, we proposed an AI system to mine CT image information to predict EGFR mutation status and mutated subtype (i.e., 19Del and L858R) and investigate the PD-L1 expression level and positive PD-L1 subtypes (PD-L1 TPS, 1%–49% and ≥50%) and further simultaneously identify both EGFR and PD-L1 status, aiming to provide support for clinical decision-making.

## Materials and Methods

### Patients Cohort and Data Collection

This study retrospectively included consecutive patients with NSCLC who visited West China Hospital of Sichuan University (Sichuan, China) from June 2019 and June 2021. The current study was performed in compliance with the Declaration of Helsinki and approved by the Institutional Review Board (IRB)/Ethics Committee. Written informed consent was waived because the data used for system development were de-identified by removing personal information. Patients who meet the following inclusion criteria were collected into this study: (1) histologically verified primary NSCLC, (2) pathological analysis of tumor tissues with thorough EGFR or PD-L1 testing results, and (3) preoperative CT images. Patients were excluded if (1) clinical data such as age, sex, and stage were missing; (2) preoperative treatment was received; (3) the duration between CT examination and subsequent surgery exceeded 1 month; or (4) tumors <1cm in size and CT imaging artifact were found. Following the screening of exclusion criteria, we selected our primary cohort (n = 3,816) for model development. Furthermore, we created a subset of EGFR cohort (n = 3,629), PD-L1 cohort (n=873), and EGFR and PD-L1 joint cohort (n = 818) who underwent staining based on surgery or biopsy specimens and gene testing (EGFR, PD-L1 or both), with the goal of evaluating the performance of our models for three prediction tasks: gene mutation status, gene subtypes, and joint prediction.

EGFR gene status was determined to be mutated (including 19Del, L858R, and Others) and wild by amplification refractory mutation system-polymerase chain reaction (ARMS-PCR) or next-generation sequencing (NGS). PD-L1 expression status was identified as positive and negative according to PD-L1 TPS (≥1% vs. <1%; TPS is the percentage of tumor cells with membranous PD-L1 staining, with TPS ≥1% indicating positivity; TPS 1%–49% and ≥50% indicating low PD-L1+ and high PD-L1+, respectively) using SP142 antibody in immunohistochemical (IHC) assays performed on the Ventana Benchmark platform. After being reviewed by senior pathologists, these gene testing results were regarded as the gold criteria in the current study. The CT data utilized in this study came from a variety of suppliers (GE, Philips, Siemens United Imaging Health) to assess the resilience of our AI system in multiple clinical contexts. All CT scans had a resolution of 512 × 512, with slice spacing ranging from 0.625 to 5 mm in the axial direction. For the electronic health records (EHRs) data collection in our study, ideally, for a unique patient, his/her EHRs data should at least include basic information, i.e., age, sex, tumor stage, and smoking status, and radiology reports in line with international standards.

For multitask AI system, we collected multimodal data that comprised (a) deep learning features based on CT images, which consisted of a global texture feature and a tumor local texture feature; (b) radiomics features that extracted and analyzed a large number of advanced quantitative image features with high throughput; and (c) clinical features that included demographics, comorbidities, and clinical symptoms.

### Data Pre-Processing

In this experiment, we obtained the training and testing cohorts from the EGFR/PD-L1 dataset by stratified and random sampling of patients at a ratio of 4:1. For the CT images, two groups of doctors were asked to delineate of the specific mask of the entire tumor. The tumor–mask pair was then fed into the radiomics model, which extracted radiomic characteristics, and the deep learning model, which extracted deep learning features. For deep learning feature analysis, a cubic region of interest (ROI) containing the entire tumor with surrounding information was supplied and retrieved local deep learning features by the local DL model, and the global DL model took the corresponding origin CT volume as input. Finally, as deep learning features, local deep feature and global deep feature were combined. Using pre-computed windowing information, all cubic ROI and origin CT volume pixels were normalized to 0–255, and all CT volumes were resized to the same size of 36 × 36 × 36 using third-order spline interpolation. To reduce overfitting, data augmentations such as horizontal flip, random resizing cropping, random rotation, and random color jittering were used throughout the training phase. For the final model training and inference, a random crop of 32 × 32 × 32 would be employed.

### Construction of AI System

**Figure 1:**
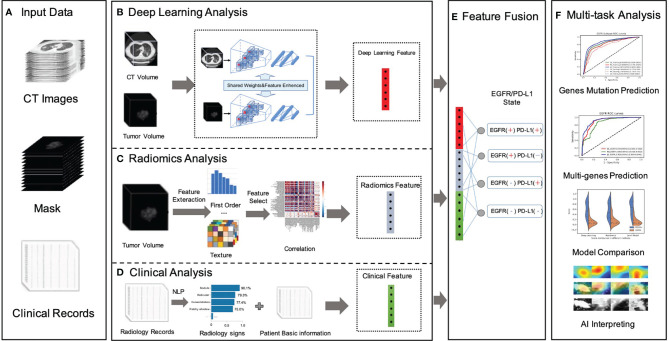
Overall workflow in our study. **(A)** Data preparation stage included original CT image data, with manually labeled tumor images, NGS testing gene mutation status, gene mutation subtypes, radiology records, and patients’ fundamental clinical indications. **(B)** A novel dual-pathway deep learning network architecture that performed CT volume and tumor volume feature extraction, named DL feature, using the trained backbone for gene prediction and further to fuse the extracted feature with another learnable pathway using an asymmetric non-local fusion module. **(C)** The pipeline of radiomics analysis model extracted radiomics features based on manually segmented contour of the tumor. **(D)** The clinical feature considered a full coverage of clinical information, including radiology signs that applied NLP techniques to extract structured labels from radiology reports. **(E)** The feature fusion utilized full connection to provide self-adaptation on the combination of deep learning features, radiomics features, and clinical features. **(F)** Validation of the proposed AI system.

We developed a deep-learning-based AI system for scalable gene prediction in patients. To summarize, our proposed AI system employed a modular pipeline method with four key components (**Figure 1**): deep learning module, radiomics module, clinical module, and feature fusion module. The following was a full description of the AI system.

For the deep learning module (**Figure 1B**), in order to pay more attention to contextual features (different lesion signs usually appear at the same time) and use these correlation lesion signs to improve the model’s representation learning ability, this study proposed a novel dual-pathway deep learning network architecture that performs CT volume and tumor volume feature as local and global information, using the weighted-share backbone to capture the dependence between tumor detail information and the whole CT information in a large range. To be more specific, both the encoders of the framework (local and global) were composed of several 3D convolution and residual blocks, and the continuous multislice (tumor and full CT images) were used to form trainable 3D data patches, which were then fed as two branch inputs to realize multiscale local and global information extraction through progressive fusion, making full use of context texture information of 3D image space. In addition, due to the different roles of global feature and local feature in specific prediction, it was necessary to conduct corresponding modeling for different extracted features; therefore, we adopted an asymmetric non-local fusion layer to implicitly modeled the attentional mechanism. For each weight-sharing branch of the backbone, for 3D volumes, we applied a 3D ResNet-18 feature extractor and fine-tuned the parameters by the pre-trained model. As a consequence, transfer learning was employed to address the issue of insufficient training data by first learning the neural network’s unique weights on the source data set. Because several gene mutations might co-exist or overlap on the same patient, a multilabel triage loss function with sigmoid active function was used instead of the standard multiclass classification loss.

The radiomics module (**Figure 1C**) retrieved and quantified a large number of characteristic data from tumor images and processed genetic and tumor information from more high-dimensional features that cannot be observed by the human naked eye to construct clinical features. The following pipeline steps were used to extract image radiomics features: (1) precise segmentation of suspected tumors, (2) extraction of large high-dimensional characteristics from suspected tumor area, and (3) filtered and reduced correlation features to prevent overfitting. To begin, 1,247 radiomics characteristics were extracted from each tumor–mask pair volume (segmented by the doctors). These characteristics included first-order (HU stats), shape, and texture properties.

The first-order feature depicted the intensity distribution of CT values in the volume of interest by common basic measures, such as mean, range, and standard deviation. The texture features were classified into five categories: (1) the gray-level co-occurrence matrix, (2) the gray-level difference matrix, (3) the gray-level run-length matrix, (4) the gray-level size-zone matrix, and (5) the gray-tone difference matrix in the neighborhood. Following that, for each feature in a specific tumor, we summarized and examined the distribution of the feature’s values across nine filters and eight wavelet transformations in high dimensions. Then, the least absolute shrinkage and selection operator (LASSO) method was used on the feature set to eliminate the correlation radiomics characteristics with low variance (<0.8). Finally, around 100-dimensional features were selected as the most useful radiomics features in LASSO model.

The clinical module (**Figure 1D**) acquired structured abnormality symptoms and the patients’ basic clinical information. Although CT imaging can provide some insight into the effectiveness of cancer immunotherapy, the clinical information of patients including age, sex, tumor staging, number, size, past recurrence, and medication status had all been linked to the efficacy of cancer immunotherapy. How to effectively combine imaging and clinical information to construct an individual prediction model remained another key problem. For the structured information, such as sex, we mainly used the one-hot strategy to convert category variables into a sparse vector space that machine learning algorithms can easily use. For the free-text reports, such as radiology record, we used the natural language processing (NLP) algorithm to perform free-text record analysis to predict patients’ radiological abnormalities into a structured label vector format (binary vector of labels for the targeting abnormality). To create uniform length vectors, the raw free texts were first vectorized using a data vectorization process. The text classifier was then trained using supervised learning, which may be used to generate labels (e.g., radiological abnormalities) automatically. The text classifier was trained using pre-annotated text–label pairs. Then, these structured symptoms label vectors and structured patients’ basic clinical information were merged into the combined vector as our clinical features.

The fusion module made use of the fully connectivity layer to provide self-adaptation based on a combination of deep learning (DL), radiomics (RA), and clinical (CL) features. Prior to the fusion action, both features were followed by a new conversion layer, specifically, a 512D-output full connection layer, which bridged the dimensional gap between the types of features and boosts the convergence of our feature fusion module. As a consequence, our model could jointly project these diverse features to an embedding feature space, allowing us to make better use of individual feature strength.

### Statistical Analysis

The following measures were used to assess the performance of our classifiers: area under the receiver operating characteristic curve (AUC), accuracy, sensitivity, and specificity. The 95% confidence intervals (CIs) for the AUC were calculated through DeLong technique. The median and interquartile range (IQR) with a 95% CI were used to represent continuous variables. Independent sample t-test was used to assess the significance of mean age of EGFR mutant and EGFR genotyping patients. The same statistical analysis was performed for scores in the PD-L1 mutant group and the PPD-L1 negative (PD-L1-) and positive (low PD-L1+; high PD-L1+) groups. χ^2^ test was used to evaluate the differences in sex and other symptoms in each cohort. The ANOVA test was used to determine whether there was a difference between the joint categories of genes mutant patients. All statistical tests were two-tailed, with statistical significance set at *P* < 0.05 considered as significant. Our implementation of the deep learning model used the Pytorch toolkit and Python 3.7.

## Results

### Patient Characteristics of Enrolled Datasets

**Figure 2:**
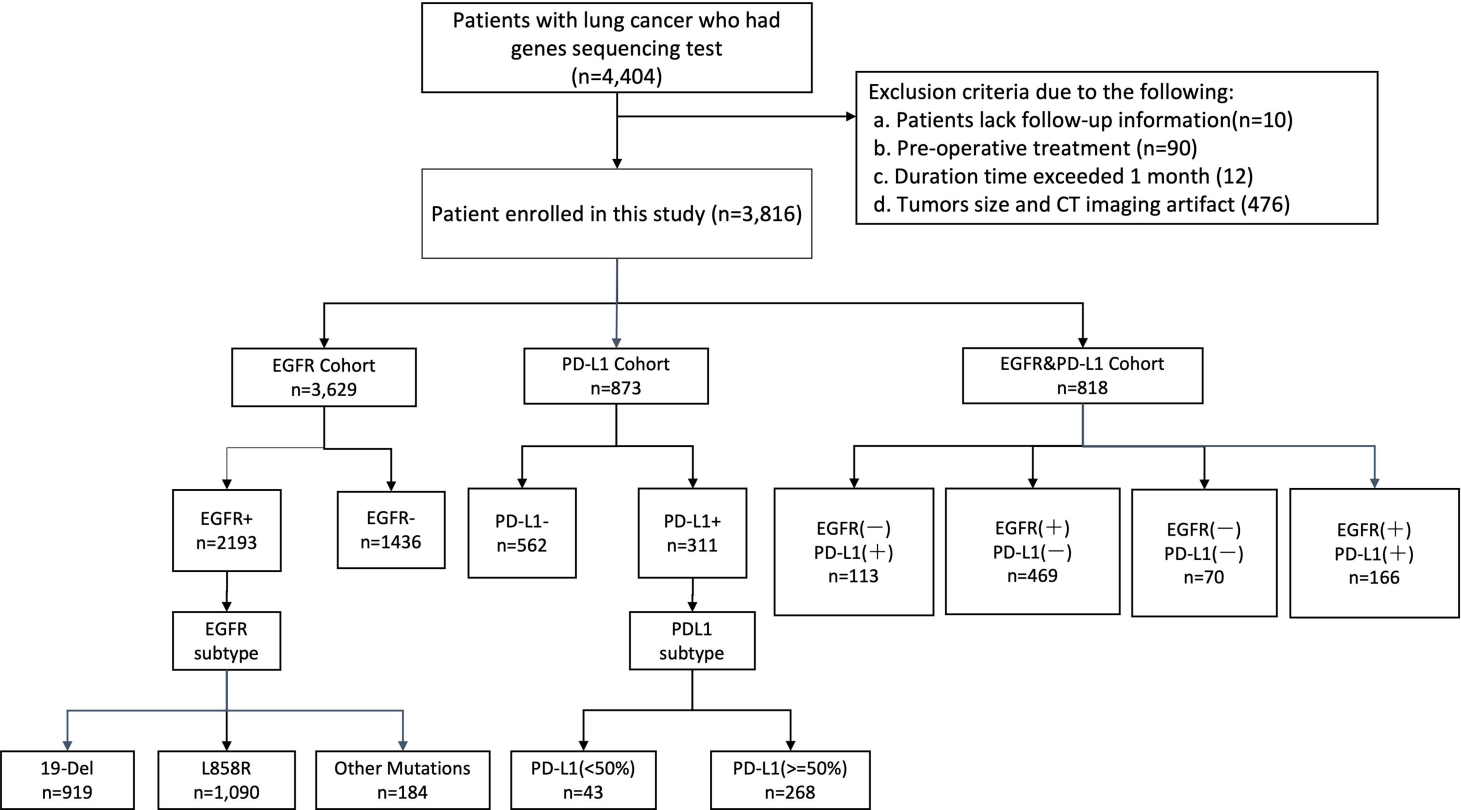
Illustration of workflow in this study. Between June 2019 and June 2021, this study included primary cohort 3,816 consecutive patients with NSCLC who visited West China Hospital (Sichuan, China) for model development and validation. Additionally, we built a subset (EGFR cohort and PD-L1 cohort) for patients who underwent staining based on histological specimens and molecular test (EGFR, PD-L1), aiming to evaluate the performance of our models on predicting events gene mutation status and gene subtype. Cohort EGFR and PD-L1 was used to evaluate model performance in joint immunity prediction.

A total of 4,404 patients were initially identified who had been pathologically diagnosed with lung cancer and had undergone the molecular (EGFR or PD-L1) test (**Figure 2**). Following eligibility screening, this study included three cohorts of 3,816 eligible patients with consecutive chest CT images. The EGFR cohort (n = 3,629), PD-L1 cohort (n = 873), and EGFR and PD-L1 cohort (n = 818) were enrolled, divided into 80% training/internal validation and 20% testing sets, to develop and optimize our AI systems for differentiating positive EGFR mutation or PD-L1 expression status from negative ones. The sum of three cohorts were not equal to the number of total cohorts due to that molecular profiling of EGFR or PD-L1 was not routinely performed for every patient. Among the whole patients, the mean age was 59 years, and 2,067 (54.17%) patients were male. There were 2,067 (54.17%) never-smokers, 3,353 (87.87%) with no family history of cancer, and 2,937 (76.97%) LUAD patients. For tumor stage, patients with stages I, II, III, and IV were 1,136 (29.77%), 284 (7.44%), 742 (19.44%), and 1,475 (38.65%), respectively. There was no significant difference for age (*p* = 0.508), sex (*p* = 0.143), smoking status (*p* = 0.759), family history of cancer (*p* = 0.503), histopathology (*p* = 0.324), tumor stage (*p* = 0.497) among these three cohorts. Demographic and clinical characteristics of included dataset are depicted in **Table 1**.

**Table 1:** Clinical characteristics of patients used to measure EGFR mutation and PD-L1 expression status.

	Total (N = 3,816, %)	EGFR (N = 3,629, %)	PD-L1 (N = 873, %)	EGFR&PD-L1 (N = 818, %)	*p-*value
**Age (years)**	59.32	59.29	58.72	58.77	0.508
**Sex, N(%)**					0.143
Male	2,067(54.17)	1,955(53.87)	471(53.95)	449(54.89)	
Female	1,749(45.83)	1,674(46.13)	402(46.05)	369(45.11)	
**Smoking status**					0.759
Current or former	1,500(39.30)	1,413(38.94)	281(32.19)	257(31.42)	
Never	2,067(54.17)	1,981(54.59)	566(64.83)	543(66.38)	
Unknown	249(6.52)	235(6.47)	26(2.98)	18(2.2)	
**Family history of cancer**					0.503
Yes	248(6.50)	236(6.50)	69(7.90)	67(8.19)	
No	3,353(87.87)	3,188(87.85)	791(90.61)	745(91.08)	
Unknown	215(5.63)	205(5.65)	13(1.49)	6(0.73)	
**Histopathology**					0.324
LUAD	2,937(76.97)	2,787(76.80)	789(90.38)	743(90.83)	
LUSC	607(15.90)	592(16.31)	47(5.38)	42(5.13)	
Other	272(7.12)	250(6.89)	37(4.24)	33(4.03)	
**Stage**					0.497
I	1,136(29.77)	1,092(30.09)	354(40.55)	347(42.42)	
II	284(7.44)	272(7.50)	68(7.79)	63(7.7)	
III	742(19.44)	700(19.29)	160(18.33)	150(18.34)	
IV	1,475(38.65)	1,402(38.63)	260(29.78)	236(28.85)	
Unknown	179(4.69)	163(4.49)	31(3.55)	22(2.69)	
**EGFR Mutation (%)**					0.934
EGFR Wild	1,436(37.63)	1,436(39.57)	–	183(22.37)	
EGFR Mutant	2,193(57.47)	2,193(60.43)	–	635(77.63)	
**PD-L1 Expression (%)**					0.639
PD-L1-	562(64.38)	–	562(64.38)	539(65.89)	
PD-L1 +	311(35.62)	–	311(35.62)	279(34.11)	
**Mutated EGFR Subtype (%)**					0.367
19Del	919(24.08)	919(24.08)	–	–	
L858R	1,090(28.56)	1,090(30.04)	–	–	
Others	184(4.82)	184(5.07)	–	–	
**Positive PD-L1 Expression (%)**					0.215
≥50%	268(7.02)	–	268(30.70)	–	
1-49%	43(1.13)	–	43(4.93)	–	

EGFR, epidermal growth factor receptor; PD-L1, programmed death ligand-1; LUAD, lung adenocarcinoma; LUSC, lung squamous cell carcinoma.

### Evaluation of Model Performance in Predicting EGFR Mutation Status

**Figure 3:**
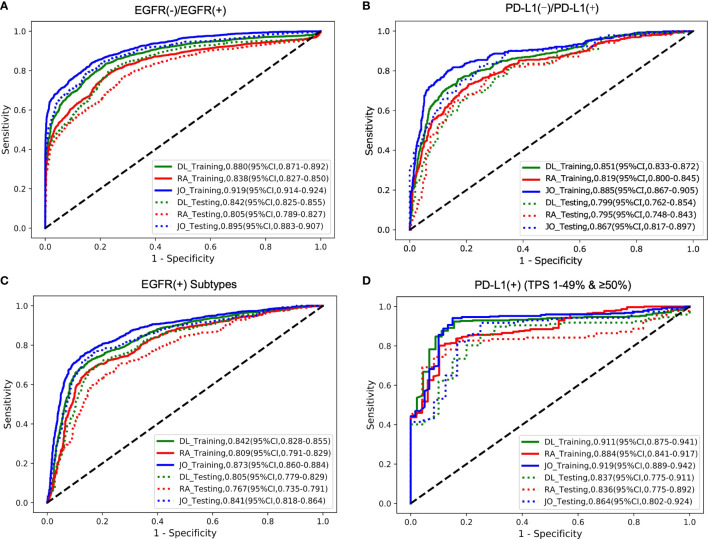
The model performances in the prediction of two outcomes (EGFR cohort and PD-L1 cohort). The ROC curves for predicting **(A)** EGFR gene mutation status (mutant or wild); **(B)** PD-L1 status (positive or negative); **(C)** EGFR gene subtype mutations (19Del; L858R or Other); **(D)** PD-L1expression status (PD-L1 TPS 1%–49% or ≥50%). DL indicated that our image-based DL system used local tumor volume and global CT volume. RA indicated that our image-based radiomics model and the JO indicated that the joint model combined with the DL feature, radiomics feature, and clinical features. The solid line represents the performances on the training set, and the dotted line represents the effect on the test set.

In this step, three models including deep learning (DL) model, radiomics (RA) model, and joint (JO) model combining the DL, RA, and CL features were trained and developed to distinguish the mutated EGFR from the wild EGFR patients and subsequently discriminate the mutated EGFR subtypes (19Del, L858R, and others). On the binary task of distinguishing mutated EGFR from wild ones, the AUCs of DL, RA, and JO models were 0.880 (95% CI, 0.871–0.892) and 0.842 (95% CI, 0.825–0.855), 0.838 (95% CI, 0.827–0.850) and 0.805 (95% CI, 0.789–0.827), and 0.919 (95% CI, 0.914-0.924) and 0.895 (95% CI, 0.883–0.907) in training and testing sets, separately (**Figure 3** and **Table 2**). Pertaining to three-way triage task discriminating the mutated EGFR subtypes, DL, RA, and JO models achieved the mean AUCs of 0.842 (95% CI, 0.828–0.855) and 0.805 (95% CI, 0.779–0.829), 0.809 (95% CI, 0.791–0.829) and 0.767 (95% CI, 0.735–0.791), and 0.873 (95% CI, 0.860–0.884) and 0.841 (95% CI, 0.818–0.864) in predicting 19Del, L858R, and other mutation status on the training and testing sets, respectively (**Figure 3** and **Table 2**). No matter which task, the proposed binary task and subtype classification, the performance of the joint model showed the best performance, and the combination of radiomics and clinical features contributed most to the EGFR prediction, which implied the associations and the complementarity of deep learning, radiomics, and clinical features.

**Table 2:** Predictive performance of EGFR status and EGFR mutated subtypes using three methods in the training and testing cohorts.

Methods	Cohorts	AUC(95%CI)	Accuracy(%)	Sensitivity(%)	Specificity(%)
**EGDFR Mutation Status**					
**DL**	Training	0.880(0.871-0.892)	0.805(0.797-0.815)	0.832(0.820-0.845)	0.783(0.769-0.798)
	Testing	0.842(0.825-0.855)	0.763(0.750-0.777)	0.797(0.777-0.818)	0.769(0.746-0.789)
**Radiomics**	Training	0.838(0.827-0.850)	0.769(0.758-0.779)	0.794(0.780-0.812)	0.760(0.743-0.771)
	Testing	0.805(0.789-0.827)	0.735(0.720-0.755)	0.768(0.748-0.793)	0.716(0.696-0.734)
**Joint**	Training	0.919(0.914-0.924)	0.840(0.831-0.850)	0.839(0.829-0.852)	0.831(0.820-0.844)
	Testing	0.895(0.883-0.907)	0.819(0.803-0.835)	0.791(0.765-0.816)	0.850(0.834-0.870)
**EGFR Subtypes**					
**DL**	Training	0.842(0.828-0.855)	0.753(0.740-0.769)	0.716(0.696-0.739)	0.853(0.836-0.873)
	Testing	0.805(0.779-0.829)	0.732(0.707-0.755)	0.707(0.676-0.742)	0.815(0.787-0.849)
**Radiomics**	Training	0.809(0.791-0.829)	0.725(0.708-0.743)	0.672(0.647-0.702)	0.848(0.830-0.870)
	Testing	0.767(0.735-0.791)	0.697(0.663-0.728)	0.705(0.670-0.745)	0.742(0.712-0.773)
**Joint**	Training	0.873(0.860-0.884)	0.790(0.776-0.804)	0.758(0.739-0.778)	0.862(0.844-0.881)
	Testing	0.841(0.818-0.864)	0.767(0.746-0.790)	0.767(0.732-0.798)	0.827(0.803-0.858)

EGFR, epidermal growth factor receptor; PD-L1, programmed death ligand-1; DL model, deep learning model.

### Evaluation of Model Performance in Predicting PD-L1 Expression Status

The trained AI system was also evaluated on the PD-L1 cohort to distinguish the positive PD-L1-positive (PD-L1 TPS ≥1%) from the PD-L1-nagetive (PD-L1 TPS <1%) patients and subsequently discriminate the PD-L1-positive subtypes (low positive PD-L1, 1%–49%; high positive PD-L1, TPS ≥50%). On the binary task of distinguishing positive PD-L1 from negative PD-L1 ones, the AUCs of DL, RA, and JO models were 0.851 (95% CI, 0.833–0.872) and 0.799 (95% CI, 0.762–0.854), 0.819 (95% CI, 0.800–0.845) and 0.795 (95% CI, 0.748–0.843), 0.885 (95% CI, 0.867–0.905) and 0.867 (95% CI, 0.817–0.897) in training and testing sets, separately (**Figure 3** and **Table 2**). Pertaining to binary task classifying the positive-PD-L1 subtypes into low positive and high positive groups, DL, RA, and JO models achieved predictive performance with the AUCs of 0.911 (95% CI, 0.875–0.941) and 0.837 (95% CI, 0.775–0.911), 0.884 (95% CI, 0.841–0.917) and 0.836 (95% CI, 0.775–0.892), and 0.919 (95% CI, 0.889–0.942) and 0.864 (95% CI, 0.802–0.924) in the training and testing sets, respectively (**Figure 3** and **Table 3**). The performance of DL model outperformed RA models in both training and testing cohorts. What is more, JO model also performed superior than both DL and RA models. Both results confirmed that the JO model was sensitive to radiomics and clinical information and differentiating positive PD-L1 from negative PD-L1 with reasonable accuracy as a diagnostic tool.

**Table 3:** Predictive performance of PD-L1 status and PD-L1 expression using three methods in the training and testing cohorts.

Methods	Cohorts	AUC (95%CI)	Accuracy (%)	Sensitivity (%)	Specificity (%)
PD-L1 Status					
**DL**	Training	0.851(0.833-0.872)	0.824(0.809-0.840)	0.758(0.729-0.791)	0.829(0.807-0.852)
	Testing	0.799(0.762-0.854)	0.770(0.727-0.800)	0.680(0.604-0.756)	0.793(0.746-0.839)
**Radiomics**	Training	0.819(0.800-0.845)	0.797(0.777-0.816)	0.732(0.688-0.775)	0.791(0.764-0.810)
	Testing	0.795(0.748-0.843)	0.759(0.717-0.797)	0.790(0.707-0.851)	0.716(0.659-0.769)
**Joint**	Training	0.885(0.867-0.905)	0.869(0.850-0.884)	0.801(0.768-0.842)	0.865(0.847-0.881)
	Testing	0.867(0.817-0.897)	0.808(0.771-0.846)	0.822(0.758-0.884)	0.752(0.714-0.810)
**Positive PD-L1 Expression with low and high PD-L1(+)**					
**DL**	Training	0.911(0.875-0.941)	0.899(0.868-0.925)	0.924(0.894-0.943)	0.844(0.750-0.940)
	Testing	0.837(0.775-0.911)	0.868(0.820-0.910)	0.857(0.816-0.905)	0.750(0.611-0.933)
**Radiomics**	Training	0.884(0.841-0.917)	0.831(0.798-0.865)	0.802(0.764-0.837)	0.898(0.811-0.963)
	Testing	0.836(0.775-0.892)	0.796(0.745-0.854)	0.744(0.672-0.809)	0.917(0.810-1.000)
**Joint**	Training	0.919(0.889-0.942)	0.917(0.894-0.938)	0.941(0.918-0.960)	0.850(0.773-0.922)
	Testing	0.864(0.802-0.924)	0.884(0.845-0.934)	0.917(0.877-0.955)	0.750(0.636-0.875)

EGFR, epidermal growth factor receptor; PD-L1, programmed death ligand-1; DL model, deep learning model.

### Evaluation of Model Performance in Predicting Both EGFR and PD-L1 Expression Status

**Figure 4:**
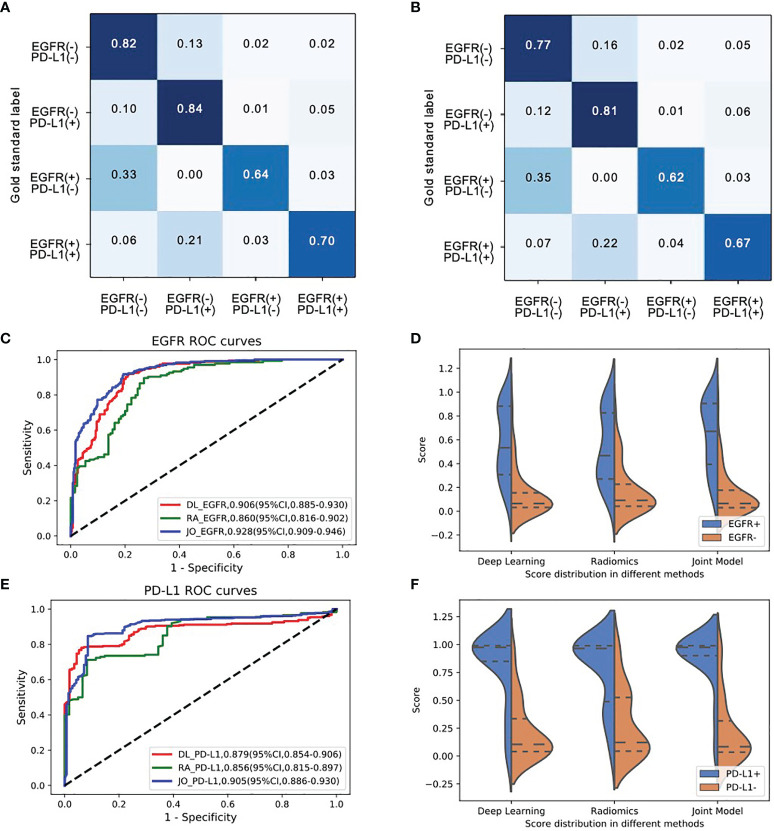
The model performances in the prediction of joint-mutant genes (EGFR and PD-L1 cohort). Confusion matrix of **(A)** training set and **(B)** testing set indicated most errors occurred in the adjacent groups. **(C)** The ROC curves and **(D)** three model scores predicting EGFR mutation; **(E)** the ROC curves and **(F)** three model scores predicting PD-L1 expression status.

We next investigated the feasibility of assessing the multigenes mutation status. Three models also demonstrated the robust performance in the co-existing immunity cohort. In the multiple task in terms of four-way classification (multitask classifier) into EGFR(+)PD-L1(+), EGFR(+)PD-L1(−), EGFR(−)PD-L1(+), and EGFR(−)PD-L1(−) groups, DL, RA, and JO model achieved the AUCs of 0.906 (95% CI, 0.885–0.930) and 0.879 (95% CI, 0.854–0.906), 0.860 (95% CI, 0.816–0.902) and 0.856 (95% CI, 0.815–0.897), and 0.928 (95% CI, 0.909–0.946) and 0.905 (95% CI, 0.886–0.930) in the training and testing sets, respectively (**Figure 4**; **Table 4**). These results proved the potential of our joint model’ ability to predict multigene events that may occur in at least two mutants on a single patient.

**Table 4:** Predictive performance of EGFR and PD-L1 status performance using three methods in the joint cohort.

Methods	Categories	AUC (95% CI)	Accuracy (%)	Sensitivity (%)	Specificity (&)
**EGFR&PD-L1 Status**					
**DL**	EGFR	0.906(0.885-0.930)	0.767(0.735-0.798)	0.920(0.884-0.954)	0.787(0.748-0.827)
	PD-L1	0.879(0.854-0.906)	0.793(0.762-0.819)	0.781(0.742-0.819)	0.939(0.904-0.969)
**Radiomics**	EGFR	0.860(0.816-0.902)	0.659(0.617-0.705)	0.896(0.856-0.944)	0.731(0.664-0.801)
	PD-L1	0.856(0.815-0.897)	0.719(0.677-0.766)	0.713(0.661-0.763)	0.918(0.864-0.972)
**Joint**	EGFR	0.928(0.909-0.946)	0.831(0.807-0.856)	0.917(0.883-0.941)	0.807(0.771-0.846)
	PD-L1	0.905(0.886-0.930)	0.848(0.825-0.874)	0.847(0.818-0.879)	0.915(0.876-0.951)

EGFR, epidermal growth factor receptor; PD-L1, programmed death ligand-1; DL model, deep learning model.

### Deep Learning Model Interpretability

**Figure 5:**
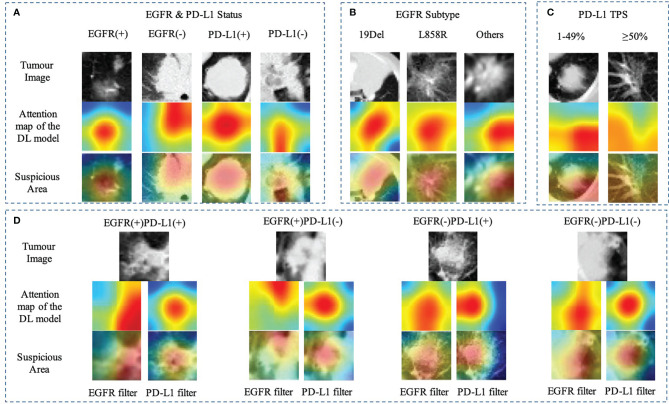
Heatmap of characteristics that contributed to the prediction of gene mutation **(A)** EGFR and PD-L1 status; **(B)** EGFR subtype (19Del, L858R, or other); **(C)** PD-L1expression status (PD-L1 TPS, 1%–49% or ≥50%); **(D)** EGFR mutation combined with PD-L1 status using different filters. The first row showed the origin tumor image in the 3D volume; the second and third rows visualize the attention regions of a network for distinct mutant categories.

For each image, the attention of the model can be visualized for human interpretability and validation. High-resolution feature visualization provides an intuitive manner to understand the distribution of features used in this investigation. The aim of this section was to evaluate and validate the potential clinical application of the joint model of heatmaps as saliency models through CT volumes. In the attention map of the deep learning model through CAM, the dark color areas might be the tumor center, visualizing the attention regions located at the border of the lesion of a network to capture the discriminative information pertaining to the prediction results of distinct mutant categories (**Figure 5**). When the deep learning model predicts gene mutation status, it could simultaneously tell human experts which area draws the attention of the model. Additionally, the deep learning framework was based on pixel-level models, with the shallow layers of the model focused on textural information between pixels in CT images, such as horizontal and diagonal edges, while ignoring some general information about the tumor. On the contrary, as the network becomes deeper, more complicated characteristics, such as tumor semantics, were learned at the deep convolutional layer. Furthermore, the radiomics model concentrates primarily on some general tumor properties rather than on specific local low-dimensional tumor aspects. As a result, for a better understanding of the joint deep learning feature, we compared the model based only on deep learning feature and the joint model feature incorporating radiomics and clinical factors on the convolution filter.

## Discussion

Accurate and rapid quantification of EGFR mutation and PD-L1 expression status is of paramount importance in identifying of NSCLC patients more suitable for EGFR-TKI or ICI therapies, further guiding clinical decision-making. However, the dynamic change in proportion of cells expressing EGFR mutation or PD-L1 level and the invasive tissue/biopsy-based nature limit the applicability of EGFR or PD-L1 testing compared to image-based assays. Thus, there is a need for a non-invasive, accurate, reliable, and reproducible method to assess EGFR/PD-L1 status. In this study, we proposed a deep learning model using non-invasive chest CT images, which demonstrated the favorable performance to predict EGFR mutation/PD-L1 expression status and their subtypes for NSCLC patients.

According to the National Comprehensive Cancer Network (NCCN) Guidelines, multiple gene status especially EGFR and PD-L1 TPS should be known before deciding whether to use either targeted therapy or immunotherapy (19). However, gene detection posed a challenge, as suitable specimens were obtained through invasive procedure. These assessments were affected by heterogeneity of antibodies, platforms, and different clinicians. For example, the current study utilized SP142 antibody to score membrane-localized PD-L1 staining in tumor cells and tumor-infiltrating immune cells, which ignored cytoplasmic- or nuclei-located PD-L1. In addition, although the treatment strategy for NSCLC has rapidly evolved with the emergence of targeted therapy and immunotherapy, persistent drug responses remain limited to a subset of patients, such as the response rates of ICIs ranged from 14% to 20% in unselected patients (20, 21). Patients with PD-L1 level ≥50% would benefit from chemoimmunotherapy than single-agent immunotherapy (response rates of 60% and 40%, respectively) (11, 22, 23). The median PFS in patients with EGFR 19Del was longer than in patients with EGFR L858R treated with EGFR-TKI (10, 24). It was worth exploring the comprehensive method to assess precise gene status.

In clinical practice, CT scans are routinely available. Frontier studies combined radiological images and deep learning technology and have become trend in screening, diagnosis, gene prediction, and prognosis of lung cancer (15, 25, 26). Previous studies proposed deep learning models trained on CT images to predict high PD-L1 expression or EGFR mutated status of NSCLC (17, 18). Meanwhile, a deep-learning model based on radiology text reports was performed to estimate objective response of PD-1 blockade in NSCLC patients (27). However, these models only focused on binary tasks of gene status constructed on single-omics data, which were unsuitable for routine clinical work. Herein, we explored an approach with promising performance to predict gene mutation and further specific type based on large sample CT images and clinical features. This detailed molecular information including EGFR mutated (19Del, L858R, other) or wild; PD-L1 (≥50%) assists physician in accurate treatment. Additionally, several studies using deep learning inferred therapeutic effects of TKIs or ICIs in NSCLS patients (18, 28, 29). We would further update this multitask AI system to predict the clinical outcomes of treatment more accurately.

In terms of algorithm, radiomics and deep learning features were integrated to mine CT image features. In addition, clinical features were integrated to try to build a prediction model with superior performance, which was more in line with routine clinical work. Not surprisingly, the performance of the integrated model was better than the deep learning model and radiomics model. This also reflected the trend of characteristic fusion. Although our AI system performed well in this aspect, it failed well short of the gold standard set by laboratory studies. To increase prediction accuracy, our AI system, for example, would benefit from other source data kinds. For example, clinical or laboratory information (such as blood biochemical analysis) might be incorporated as an additional information source to our joint AI system.

Our study has some limitations. First, this was a single-center study, and the predictive value of our model still needs to be validated in other medical centers. Second, this was a retrospective study. NSCLC patients may take multiple genes detection during treatment, which may cause some selection biases. Finally, this research only focused on EGFR and PD-L1. More extensive data would be collected to support additional mutation of NSCLC, such as ALK, ROS1, and KRAS mutation in the future.

In conclusion, this study demonstrated an AI system’s value in assisting medical professionals provide a non-invasive and easy-to-use method to identify the expression status of common genes EGFR and PD-L1 through CT images, which may serve as a predictive biomarker for guiding the target therapy and immunotherapy in NSCLC patients. Future refinement and improvement will expand its use into predicting other common genes mutation in larger and prospective trials.

## Data Availability Statement

The raw data supporting the conclusions of this article will be made available by the authors, without undue reservation.

## Ethics Statement

The studies involving human participants were reviewed and approved by the Institutional Review Board (IRB)/Ethics Committee of West China Hospital. The patients/participants provided their written informed consent to participate in this study.

## Author Contributions

WL and YY were involved in the study design. CW, JM, and JS were involved in the organization of the entire project, data analysis with a clinical perspective, and manuscript writing. CW and JS collected the imaging and clinical data. JM, SZ, and ZL were involved in the establishment of the algorithm. All authors contributed to the article and approved the submitted version.

## Funding

The study was supported by National Natural Science Foundation of China (82100119, 91859203, and 81871890), the Science and Technology Project of Sichuan (2020YFG0473), Chinese Postdoctoral Science Foundation (2021M692309), Postdoctoral Program of Sichuan University (2021SCU12018), and the Science and Technology Achievements Transformation Foundation and Postdoctoral Program of West China Hospital, Sichuan University (CGZH21009 and 2020HXBH084).

## Conflict of Interest

The authors declare that the research was conducted in the absence of any commercial or financial relationships that could be construed as a potential conflict of interest.

## Publisher’s Note

All claims expressed in this article are solely those of the authors and do not necessarily represent those of their affiliated organizations, or those of the publisher, the editors and the reviewers. Any product that may be evaluated in this article, or claim that may be made by its manufacturer, is not guaranteed or endorsed by the publisher.
